# The impact of user characteristics of smallholder farmers on user experiences with collaborative map applications

**DOI:** 10.1371/journal.pone.0264426

**Published:** 2022-03-02

**Authors:** Mona Bartling, Anthony C. Robinson, Harold Achicanoy Estrella, Anton Eitzinger

**Affiliations:** 1 Department of Geoinformatics – Z_GIS, University of Salzburg, Salzburg, Austria; 2 Department of Geography, The Pennsylvania State University, State College, Pennsylvania, United States of America; 3 Alliance of Bioversity International and CIAT, Cali, Colombia; National Taiwan University, TAIWAN

## Abstract

In the future, farmers will have increasing opportunities to use collaborative smartphone applications for agricultural management. Geospatial information in combination with agricultural-relevant information is a great source of knowledge for farmers. Including maps in collaborative mobile agriculture applications benefits communication processes related to agricultural-relevant questions. Ensuring a positive user experience with map interfaces depends on their design. To develop design guidelines for map-oriented mobile agricultural applications, this study evaluates 24 different map design variations (varying in their elements and degrees of complexity) and characterizes their user experience with 72 coffee farmers as study participants. Our findings show that the most crucial factors for a positive user experience were restricted interactivity, simple tasks to conduct (selecting single point features), and a simplified base map style, highlighting relevant landmarks. Since our farmers consisted primarily of less-experienced smartphone and map users, our findings may also be helpful for users in general, sharing similar user characteristics. While empirical, in-situ studies pose many challenges, they provide relevant insights into the real use situation and user behavior of mobile map applications. Our findings help establish some basic principles for designing map adaptations, serving as a guideline for creating effective mapping applications, which adapt to the farmers’ contextual factors. Based on our study results, we suggest future research for continuing conceptualizing principles of map design adaptation and support this effort through empirical, in-situ studies for relating contextual user factors to the adaptation behavior of map applications.

## 1. Introduction

Given the anticipated global population growth (9.7 billion people to 2050 [1]), the agricultural industry faces challenges for coping with the need for an estimated 70% increase in food production. Agriculture is of great importance for the gross domestic product (GDP) of many nations and amounts to one-third of the GDP in Sub-Saharan Africa [2]. Therefore, it is necessary to find measures to contribute to the production activity of the agricultural sector in Africa.

Since the beginning of organized agriculture, farmers have always communicated with each other about planting strategies, plant seeds, and land property issues [3]. Information and Communications Technology (ICT), including mobile phones, tablets, and smart applications, are essential tools for communication purposes to support daily routines and for enabling collaborative participation platforms. With ICT tools, farmer-to-farmer communication is now possible to conduct via the internet and, therefore, has the potential to extend the geospatial reach of these processes. ICT tools could improve agricultural productivity in this extended communication radius [4] and enable direct communication with farmers, making it possible to collect data on local conditions and phenomena. Besides, socially, economically, and locally marginalized farmer (communities), for example, indigenous or female farmers, can be integrated into agricultural extension services to enhance the sustainable implementation of new methods, techniques, and technology [5]. The potential advantage of using ICT tools to support agriculture lies in the cost-effectiveness, simplicity, and the possibility to exchange data, information, and knowledge in a fast, dynamic, and interactive way for linking different participation stakeholders [2, 4, 6].

Communication, collaboration, and participation approaches of agricultural ICT tools have also been combined with the geospatial domain [7]. The benefits of using collaborative and participatory maps to link communities and involve the public in decision-making processes have been widely discussed (see for example Atzmanstorfer and Blaschke [8], Sieber [9], Brown and Kyttä [10], and Steiniger, Poorazizi, and Hunter [11]). In principle, participatory and collaborative mapping is the creation of maps by non-expert users to represent local, geospatial knowledge and information by using cartographic methods [12]. The application of participatory and collaborative mapping tools is diverse and can be used in domains such as community development and urban, regional, environmental, and agricultural planning [10]. For the agricultural domain specifically, Eitzinger et al. [13], Eitzinger et al. [14], Corbett [12], Braslow, Cordingley, and Snyder [15], and Xiang and He [16] discuss the integration of map elements in collaborative, agricultural tools with the goal to express and visualize local, geospatial knowledge, relevant for the agricultural productivity of the farmers, land and resource planning, etc. Including map elements in collaborative, agricultural tools provide the crucial benefit to geo-reference information (e.g., on crop distribution or delineation of agricultural land) provided by the users and subsequently enables user interactions with these geospatial data [13, 14].

For the design of ICT tools for farmers, Aker et al. [4] highlight problematic issues regarding the usability of such tools. An effective agricultural application interface should respond to low (digital) literate or (digital) illiterate users. In complementary work, Adegbidi et al. [17] propose five variables that are of particular importance for influencing the adoption of ICT tools by farmers: self-efficacy, perceived usefulness, perceived ease of use, subjective norm compatibility, and job relevance. Here, the variable perceived ease of use again relates to the previously mentioned usability of applications.

Therefore, in research about map applications used for collaborative or participatory approaches, one important aspect to draw attention to is usability. As the ISO 9241–11 standard for usability describes [18], it is essential for designing a usable product to define the target user, the system goals, and the context of use. However, a conflict between user needs and design priorities can occur when collaborative agricultural map applications are intended to be useful to a broad user audience, consisting of farmer communities of multiple regions, extension workers, or scientists. In practice, this may lead to significant usability issues.

In a previous usability study on a map-oriented collaborative application for citizen participation, Bartling et al. [19] evaluated usability with users that differed significantly in their experience with smartphones. The results of this prior study showed that less experienced smartphone users faced greater usability hurdles when interacting with the collaborative mapping application. For these users, the interactivity complexity and range of functionalities can be simplified to respond to the cognitive and perceptual limits of the users [20]. As the range of features and possible interactions in a contemporary mobile map application is very wide, we need to know how to properly adapt these functions and the details shown in a mobile mapping application (i.e., the map design) to specific user audiences. This is necessary to avoid usability disadvantages for particular user groups.

The goal of our present study, therefore, is to understand how to adjust the map design of an agricultural collaborative mapping application to the characteristics (also regarded as context) of farmers and to characterize which contextual information provided in the mobile map interface shapes usability when interacting with the application. In addition to usability, we also want to characterize the user experience (UX), which the ISO standard 9241–210 defines as “user’s perceptions and responses that result from the use and/or anticipated use of a system, product or service” [18]. Common usability and UX evaluation metrics are task success, efficiency, learnability, memorability, and user satisfaction [21]. To analyze usability and UX in our study, we particularly focused on evaluating the task success, participants’ comfort and confidence. Our research effort contextualizes within recent trends to evaluate the relevance and impact of specific user context attributes on map design UX [22, 23].

In this article, we evaluate the adaptive map design affordances of a collaborative mobile mapping application designed for agricultural users. Our study characterizes and compares different approaches for implementing mapping interfaces to support agricultural users. For our study design, we sought to answer the following research questions to understand the influence of user context on map design UX: (1) how do map elements relate to the task success in users’ map activities and user contexts?; and (2) how do map elements modify participants’ comfort and confidence? To answer these questions, we created 24 map design variations, consisting of three different map-reading tasks, base map styles, and interactivity variants. Our evaluation was conducted through two case studies via digital field surveys and engaged with a total of 72 coffee farmers in the Mt. Elgon region of Uganda and the Popayan region in Colombia.

In the sections that follow, we begin by reviewing related work where we discuss concepts of adjusting the map design to user context in mobile map applications. As our two case studies differed slightly, we present our research methodology and results independently for each experiment in Uganda and Colombia. We will then discuss the findings from both case studies. To conclude, we synthesize our findings, discuss their impact, and explore the potential for future research in user context-based map design adaptation based on what we have learned.

## 2. Related work

The adaptation of interface elements within the domain of GIScience is identified using several terms, including adaptive maps, context-aware maps, and personalized maps. Each of these terms refers to a map that adjusts to the user context [24–26]. Adaptive or personalized interfaces are useful for reducing the cognitive load of users or when a user’s device has limited speed or storage capacity, filtering for user-relevant content [24]. Here, the data, their visualization (communication of information), and the interface elements (for carrying out map-related activities) can be subject to adaptation [27, 28]. An adaptation can then improve the UX to increase user satisfaction, which may improve the capability for decision-making processes since users can be sufficiently informed [29]. Enhanced UX can have a particularly strong impact on mobile devices, where users need to make fast decisions [26]. Numerous websites and applications are already applying concepts of context-aware systems (e.g., recommendations systems); among these is Google Maps, which utilizes users’ locations for personalization [30, 31] and monitors search behavior to adapt the map content.

Users on a mobile device generally deal with a significant cognitive load, having to work with a smaller screen size (and therefore smaller visualizations) and reduced device capacities (such as battery status or internet connection) [26]. Adjusting the map design to this contextual information may substantially aid the users in avoiding information overload. In general, the context of use may differ substantially between users. Hence, the context to take into account for the design of map interfaces should be comprehensively reviewed. Petit et al. [25] offer three categories for structuring context: the user context (“who”, e.g., the users’ profile), the geographical context (“what”, e.g., the location and its properties), and the appliance context (“how”, e.g., the computing system, technical aspects of the application). As Kubíček et al. [29] argue, it is also essential to consider the purpose of the application and the activities that a user wants to carry out. The same authors discuss the importance of understanding how users perceive reality to understand in detail their preferences and needs. Understanding users’ interests can be achieved by analyzing activities in the past, e.g., information that the user accessed. The acquired information can then be used to recommend related content for the future [24, 32]. However, as Raubal and Panov [26] state, understanding and modeling users’ characteristics, such as interests and abilities, is challenging. Therefore, an adaptation model needs to consider and analyze multifold feedback channels, which define the user, for extracting patterns.

An adaptation model forms the basis for creating adaptation and recommendation rules for the map design [31]. For example, certain interface elements (e.g., a service) appear only when the user is located in a specific area or information is adapted based on users’ preferences [33] or digital and spatial literacy levels. Depending on the user context, the level of detail of (spatial) information, the classification, the information filtration, the access to interface elements, color schemes, symbols, or the default map position can also be subject to adaption [27,30].

## 3. Experiment 1: Ugandan case study

As our study objective was to evaluate different ways of implementing and adjusting the design of mapping interfaces for agricultural users, we carried out two case studies to evaluate the UX of different types of map design variations. To respond to our posed research question and evaluate the impact of user context on map design UX of different approaches for implementing agricultural collaborative mapping tools, we created a digital field survey to test 24 map design variations. These variations were a combination of different map-reading tasks, base map styles, and interactivity variants. The study design (for both experiments) was reviewed and approved by the CIAT Institutional Review Board (#2019-IRB8). All participants gave informed and written consent.

In a first experiment, we tested these variations with coffee farmers of the Mt. Elgon region in Uganda. For the purpose of not overburdening the participants by presenting to them all of the 24 map design variations, we applied a between-subjects study design where we assigned a randomized subset of 12 map design variations in random order to each participant. Based on pre-tests, we chose 12 map design variations to target a suitable survey completion time.

The recruited number of participants was selected in such a way that each map design variation was used at least 15 times. We collected statistics on the task success and participants’ self-rated comfort and confidence in using the map design variations. We further collected information on participant characteristics to contextualize these findings.

**Fig 1:**
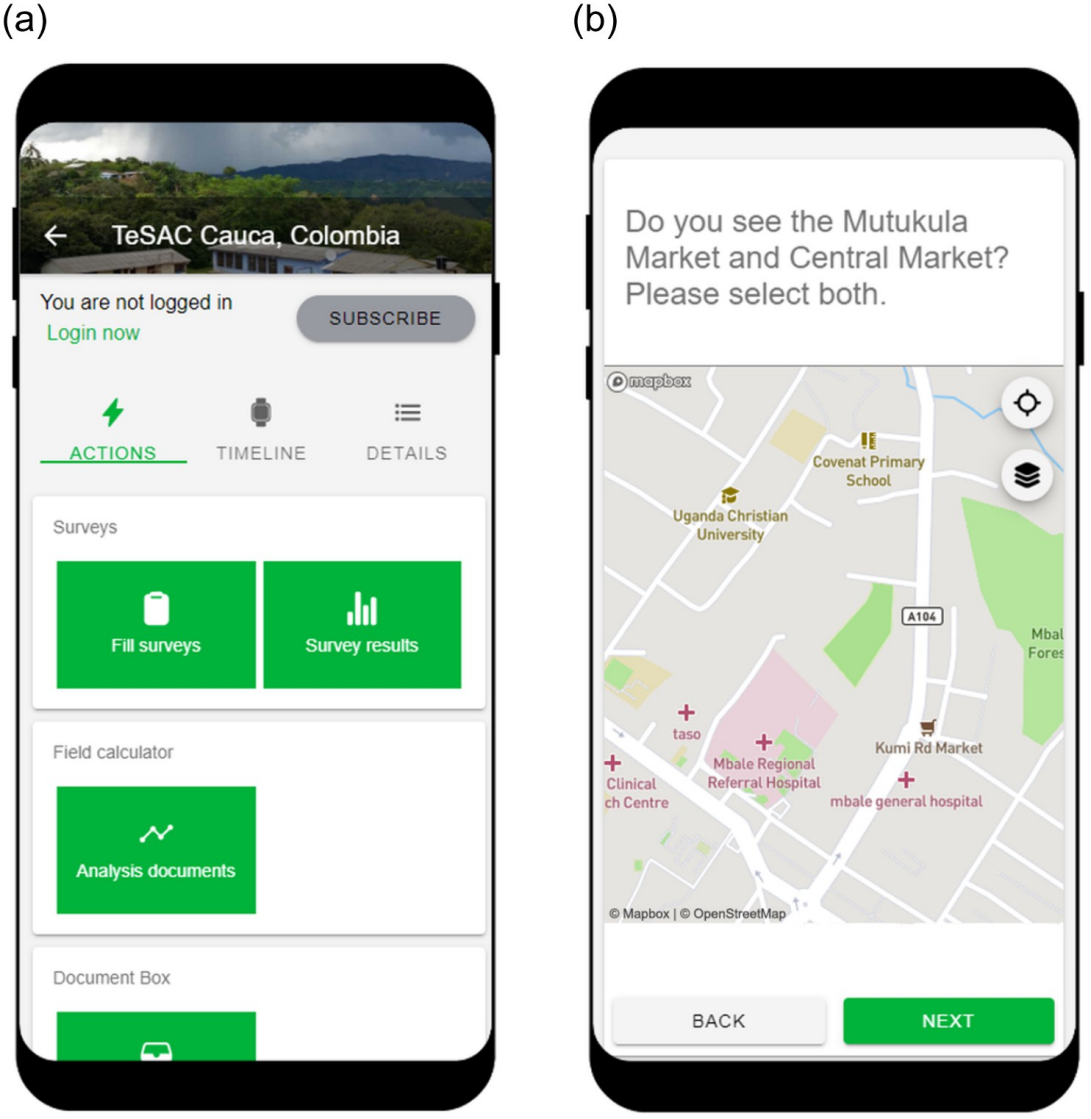
GeoFarmer application (note that permission to use the figure was obtained from Fundación CEC; also note that the figure uses map data from Mapbox and OpenStreetMap and their data sources; see mapbox.com/about/maps and openstreetmap.org/copyright).

We created the survey with the GeoFarmer application (home.geofarmer.org; see Fig 1 for a screenshot of the application and the survey module).

In the sections that follow, we first describe the study design and then continue to present the findings and observations of this first experiment.

### 3.1 Participant recruitment

For the Ugandan Case Study, the participants were recruited through collaborating with the Olam regional office in Budadiri, Uganda. Olam is an international agricultural company that buys, processes, and sells coffee in the regional office in Budadiri and works together with coffee farmers in the region. Participant recruiting was done through phone contacts with Olam-collaborating farmers.

We asked to invite at least 35 female and male farmers ranging from ages 18 to 65 years, with English reading and writing literacy and familiarity with smartphones. We especially encouraged female farmer participation to ensure equal gender representation.

In total, 37 participants were recruited, from which two participants did not finish the survey due to technical issues. The mean age was 38 (SD = 11.58). Seven women and 28 men participated. Unfortunately, in the Budadiri region more male farmers than females were enlisted in the Olam program, which is why our survey recruiting failed to achieve a gender balance. Further, 51.4% of all participants were owners of a smartphone, though all participants had previously used smartphones. How comfortable the participants felt using a smartphone was rated on a 5-point Likert scale (where low values equaled less comfort), with a resulting mean of 3.89 (SD = 1.53). The same was conducted with map use comfort, leading to a mean of 3.6 (SD = 1.17). Table 1 shows a detailed view of the participants’ profiles.

**Table 1:** Participants’ characteristics.

Age	Mean: 38.29
	SD: 11.58
	Min: 18
	Max: 71
Gender	Female: 7
	Male: 28
Education	Primary school: 3
	Secondary school: 17
	Undergraduate: 9
	Postgraduate: 5
	Other: 1
Smartphone ownership	Yes: 51.4%
	No: 48.6%
How comfortable participants felt using a smartphone 1 (min)—5 (max)	Mean: 3.89
	SD: 1.53
How frequently participants used a smartphone 1 (min)—3 (max)	Mean: 2.46
	SD: 0.7
Whether participants used the smartphone for applications other than Social Media	Yes: 60%
	No: 40%
Whether participants had prior experiences with using a map	Yes: 60%
	No: 40%
How comfortable participants felt using a map 1 (min)—5 (max)	Mean: 3.6
	SD: 1.17

### 3.2 Materials

We created 24 map design variations that were a combination of three different map-reading tasks, three base map styles, and three interactivity variants (Table 2).

**Table 2:** Map variation categories.

Map-reading tasks:	Base map styles:	Interactivity variants:
T1: Selecting single point feature	Landmark map Simple map Mapbox Streets	Static map Restricted map (extent + zooming) Non-restricted map
T2: Selecting multiple point features	Landmark map Simple map Mapbox Streets	Static map Restricted map (extent + zooming) Non-restricted map
T3: Sketching	Landmark map Simple map Mapbox Streets	Static map Non-restricted map

The first two map-reading tasks are basic cartographic interaction primitives focusing on visual searches on the map [34–36], where participants had to find and select single or multiple point features. For a third map-reading task variant, and following a typical participatory mapping task [12], we asked participants to sketch on the map to draw and highlight boundaries and areas. These types of map-reading tasks are typically found in participatory and collaborative tools and can be used, for the agricultural domain, to upload information on market accessibility (e.g., providing information on frequently accessed markets), land use (e.g., uploading information on crop distribution), agricultural practices (e.g., selecting optimal places to plant shade trees or locate irrigation systems), characteristics on farm land (e.g., delineating natural resources or aspects on local territories), etc. Each map-reading task featured a question to prompt participants’ interaction (see S1 Table for each question) and a combination of map design elements (base map style and interactivity variant).

**Fig 2:**
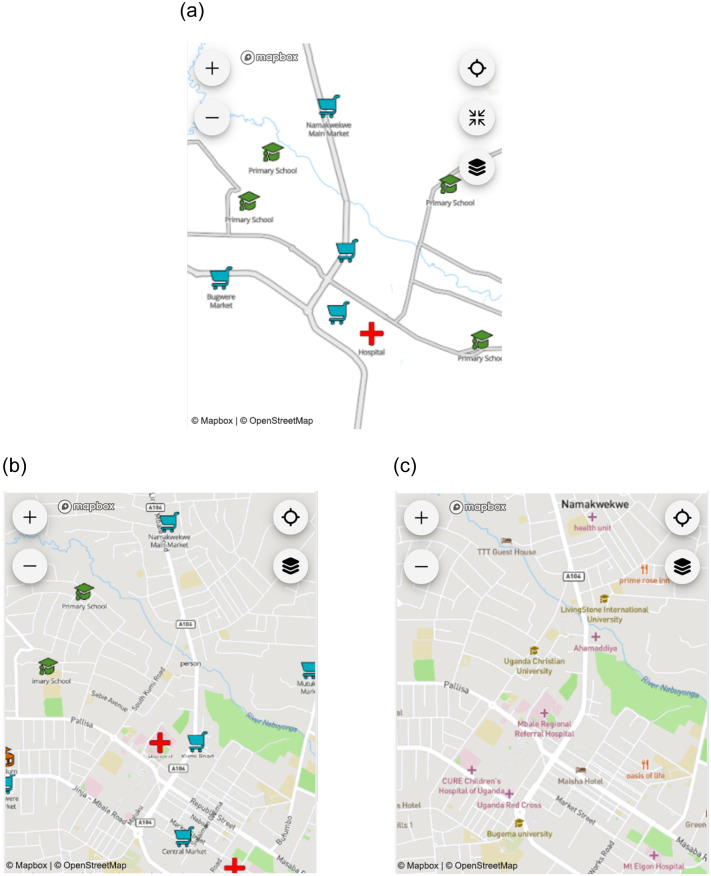
Types of base maps with a) Landmark map, b) Simple map, and c) Mapbox Streets base map (note that the figures use map data from Mapbox and OpenStreetMap and their data sources; also see mapbox.com/about/maps and openstreetmap.org/copyright).

Following approaches by Konečný et al. [37], Bestgen et al. [38], Edler et al. [39], and Liao et al. [35], we tested three different versions for the base maps. The base map styles differed in terms of their symbology and quantity of information, which potentially impacts the cognitive load of the participants and influences the usability evaluation. Each base map was created with Mapbox and focused on the town Mbale, Uganda. First, we provided a landmark-based map, where relevant landmarks were used for orientation, and only a very simplified base map was provided, visualizing only the most basic infrastructure (e.g., main streets and natural landmarks; see Fig 2a). The landmarks were identified through local contact partners to present locally most relevant landmarks. We used the Mapbox Maki icons (labs.mapbox.com/maki-icons) and adjusted color and size to highlight the landmarks.

For the second base map variant, we adjusted the Mapbox Streets base map and removed the Mapbox-default points of interests, and only visualized the primary and secondary streets. We used the landmarks from the first base map variant and applied them to this simplified Mapbox Streets base map (Fig 2b). For the third base map variation, we used the Mapbox Streets base map without applying any changes to it (Fig 2c).

Additionally, we varied the degree of interactivity to test the effect on the usability evaluation of varying interface complexities [20]. For the first two map-reading tasks (selecting single and multiple point features) we applied three variations: first, participants were presented a static map without zooming and panning. For the second variant, participants were allowed to zoom and pan within defined bounds. For the third variant, no restrictions were applied and the participants were allowed to navigate freely on the map.

For the third map-reading task (sketching), two variations were presented: for the first variant, participants received a static map and a limited set of sketching functionalities. For the second variant, participants had to navigate to the area of interest to draw on and were provided with a full set of sketching functionalities in the interface.

### 3.3 Survey structure and procedure

The survey consisted of three parts. In the first part, we provided instructions on how to solve the map-reading tasks of the survey. For that, we provided tutorial videos that explained how to use the different elements of the maps. These videos covered:

- how to move the map by zooming and panning,
- how to select point features on the map, and
- how to sketch on the map.

After the short instruction section, the second survey part featured a subset of the 24 map design variations. As we applied a between-subjects study design, each participant received a randomized subset of twelve map design variations. After each map design variation, we asked the participants to self-report their comfort in solving the map-reading tasks and their confidence in the correctness of their solution to the map-reading task. We used a five-point Likert scale (strongly disagree to strongly agree) to the following statements:

- I felt comfortable answering the question.
- I am sure that my answer is correct.

In the last part of the survey, all participants were asked questions about their preferences for the three different base map styles (Landmark map, Simple map, and Mapbox Streets). We presented each base map style individually by providing a screenshot and used a subset of the System Usability Scale (SUS) [40] with a 5-point Likert scale for measurement (strongly disagree to strongly agree) to respond to the following statements:

- I think that I would like to use this map frequently.
- I think the map is easy to use.
- I would imagine that most people would learn how to use this map very quickly.

We then posed three questions comparing the Landmark map with the Simple map, the Simple map with Mapbox Streets base map, and the Landmark map with the Mapbox Streets base map. For each comparison, we asked the participants to select the base map they preferred or to indicate if they liked both equally.

The procedure of the surveys was as follows: groups of 8–10 participants were given a time slot for the survey. This way, multiple participants were carrying out the survey simultaneously. We conducted the survey in an office space in the Mt. Elgon Olam regional office in Budadiri, Uganda. We welcomed the participants, asked them to sit at a desk, introduced them to the topic and general purpose of the survey and its procedure, and handed out smartphones with the survey displayed. The used smartphones were Samsung Galaxy A3 (2017) with a 4.7” display using Android version 7. After having finished the survey (which ranged between 30 and 45 minutes per participant), the participants returned the smartphones and received monetary compensation (15,000 USh).

### 3.4 Data analysis

In a first step, we calculated descriptive statistics regarding the task success and participants’ comfort and confidence when interacting with the different map design variations. Next, for a deeper statistical understanding of the relation between the task success, participants’ comfort and confidence, map design variations, and user characteristics, we applied a logistic mixed-effects model [41]. In a regression model with only fixed-effects variables, all independent variables are regarded as independent and on the same hierarchical level. However, in our case, we took multiple measures per participant and map design variation. Hence, these multiple responses per participant and map design variation cannot be regarded as independent from each other and are therefore hierarchical. These structures can be incorporated through random effects.

We used the glmer-function available in the lme4 package (github.com/lme4/lme4) for R to build the models. Since all categorical variables have an order, we converted these variables into numerical values. Even though this procedure prevents the interpretation of the coefficient, it is possible to evaluate the linear effect (negative/positive) between the variables.

We built three models where we varied the dependent variable and converted each to binary: task success (successful vs unsuccessful), participants’ comfort (comfortable vs uncomfortable), and participants’ confidence (confident vs unconfident). As predictor (independent) variables, we inserted the remaining variables. As random effects, we determined the participant and map design variation identifiers as random intercept since the map design variations are applied repeatedly and participants have a different average effect over their response to them (as described above). To understand the effect of each variable, we evaluated the p-value and the standardized beta coefficient (SBC).

### 3.5 Results

In the following, we will present the results of the descriptive analysis and the regression model. Through cross-validating the responses to the participants’ comfort and confidence questions (posed after each map design variation), we detected a strong response bias. Hence, for analyzing the survey results of the Ugandan experiment, we opted to not include these data in our analysis (see Observations-section for more detail). We will further reflect on the detected bias in the Discussion-section.

#### 3.5.1 Task success

The average time each map design variation was used amounts to 17.5 times (see also the frequency of each map design variation in S1 Table). The mean task success amounts to 51%. Ideally, the task success should be 100%. However, as Nielsen [42] depicts, most websites have a task success lower than 50%. Specifically to the map application domain, Skarlatidou and Haklay [43] analyzed different platforms, resulting in a task success ranging between 64% (MapQuest) and 83% (Google Maps). However, the calculation of these task success scores also included partial task success, which we did not include in this study.

**Fig 3:**
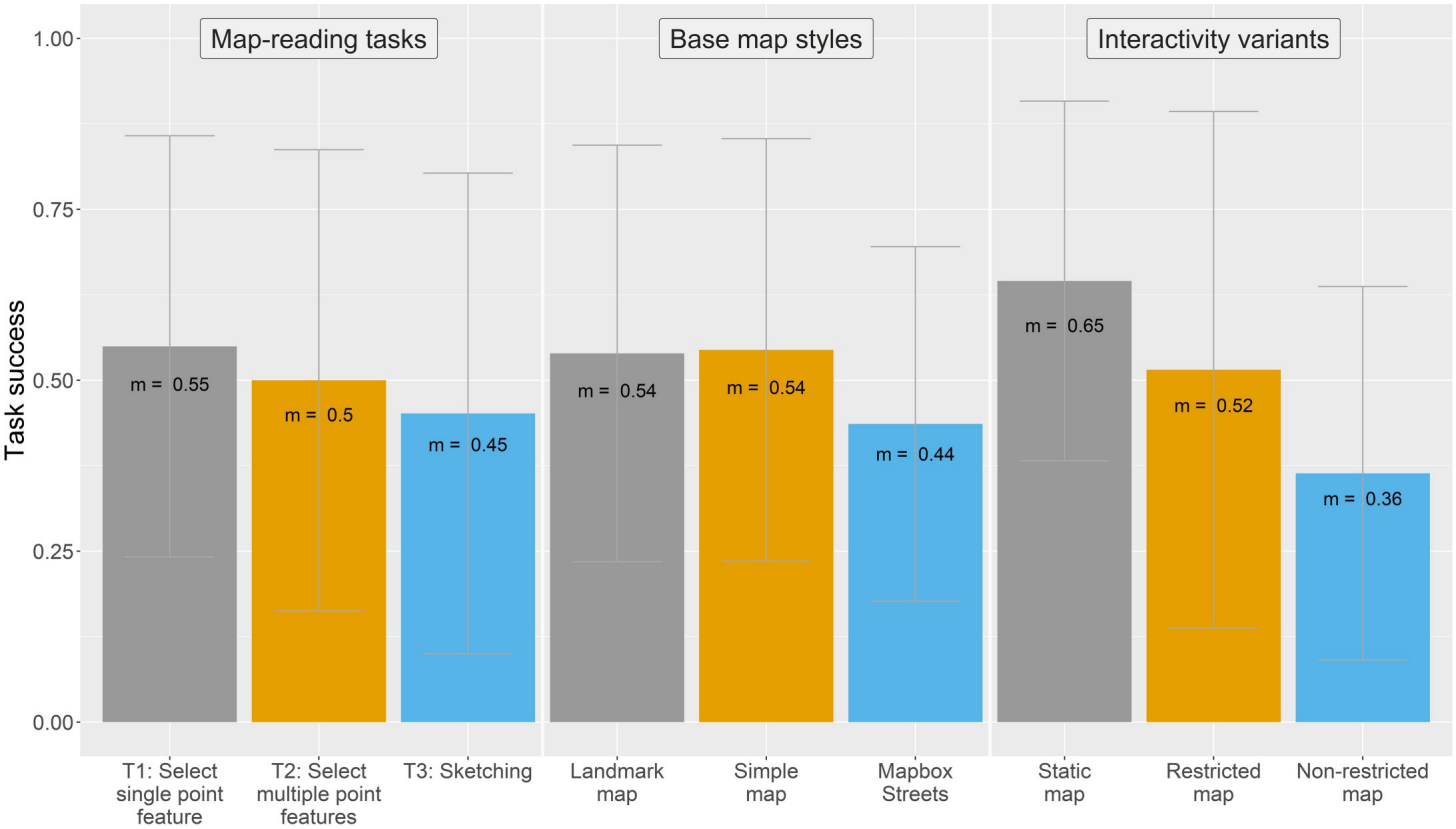
Task success of map-reading tasks, base map styles, and interactivity variants.

For understanding the individual task success of the different map design variations, Fig 3 depicts these in a more detailed way, differentiating between map-reading tasks, base map styles, and interactivity variants.

Generally, the mean task success decreases in each plot reading the individual bars from left to right. The map-reading task to select a single point feature (T1) yields the highest task success (55%). For the base maps, the Landmark map and the Simple map had a higher task success (both with 54%) than the Mapbox Streets base map (44%). A much greater difference in the scores is visible for the interactivity variant. Here, the static map yields with 65% the highest scores compared to the other two variants (52% and 36% for the restricted and non-restricted version, respectively).

**Fig 4:**
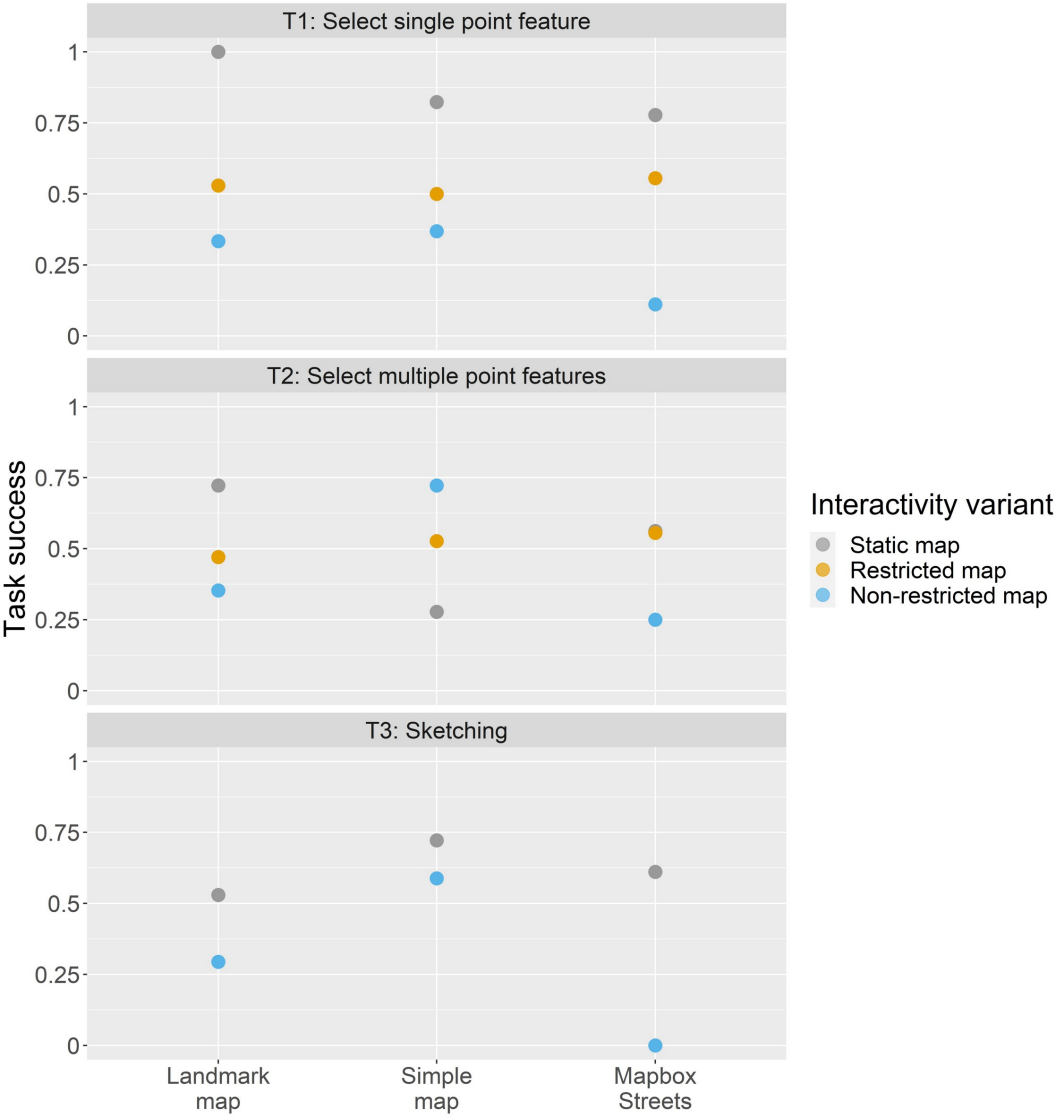
Task success of map-reading tasks related to base map styles and interactivity variants.

To analyze the task success of the map-reading tasks in relation to the other map design elements, we calculated result scores for each of the 24 map design variations (Fig 4). Mostly, the map design variations with the highest task success are those that are coupled with a static map. Having a look at the specific map-reading tasks, the map-reading task to select single point features (T1) was predominantly aided when coupled with a static Landmark map. In contrast, the map-reading task to select multiple point features (T2) worked also best with a static Landmark map but also yields high task success scores for a non-restricted Simple map. The map-reading task where we asked participants to sketch on the map (T3) was aided when complemented with a static Simple map. In terms of the base map styles, we observe high task success scores for all base maps when coupled with a static map interface. However, for map-reading task T2 (select multiple point features) the Simple map and Mapbox Streets base map yield higher scores for the non-restricted and restricted map version, respectively. We further detected statistically significant correlations for map-reading task T1, where the task success rate of all base map styles was negatively impacted by higher degrees of interactivity complexities (p < 0.001 *** for the Landmark map, p < 0.05 * for the Simple map, and p < 0.01 ** for the Mapbox Streets base map). For map-reading task T2, while the task success of the Landmark map was negatively impacted by larger interactivity complexities (p < 0.05 *), the task success of the Simple map positively correlated with larger interactivity complexities (p < 0.01 **). No statistically significant relationships were found for the task success of the Mapbox Streets base map in combination with map-reading task T2 and any base map style in combination with map-reading task T3.

#### 3.5.2 Logistic mixed-effects regression analysis

For analyzing the relation between user characteristics, map design elements, and task success, we ran a logistic mixed-effects regression analysis. Table 3 shows the SBC together with the p-values of each variable. The interactivity variants are shown as highly significant (p < 0.001 ***) with a negative relation. Hence, higher freedom in interactivity relates to a decrease in task success. For a higher interactivity complexity, participants had to zoom and pan to the respective location/extent on the map, which required more digital and spatial skills. Although only marginally significant with p < 0.1, the use of applications other than social media had a positive effect on the task success. The SBC reveals the same pattern and determines both variables as most impacting on the dependent variable.

**Table 3:** Regression model with task success as the dependent variable (see S3 Table for odds ratios).

#	Variables	SBC and p-value
1	Map-reading tasks	-0.57
2	Base map styles	-0.50
3	Interactivity variants	-1.33 ***
4	Time spent on task	-0.36
5	Age	-0.53
6	Gender (male/female)	0.51
7	Education	-0.26
8	Owner of smartphone (yes/no)	0.04
9	Smartphone use comfort	-0.06
10	Smartphone use frequency	-0.58
11	Smartphone application use other than social media (yes/no)	0.95.
12	Map use experience (yes/no)	0.06
13	Map use comfort	0.23

#### 3.5.3 Observations

Throughout the administration of our experiment in Uganda, a substantial portion of participants asked the survey team to clarify some issues. The main issues that we observed related to understanding map-reading tasks and instructions, understanding the English language, eyesight constraints, and user-reported inexperience with smartphone use. This has led the survey team to intervene and help by clarifying aspects on the survey texts and map-reading tasks, which might have affected how participants performed in the survey. We also observed a very strong response bias for the self-rated map and smartphone use experience of the participants, where many participants selected “comfortable” even though many were clearly not comfortable. The same was observed for the opinion and preference questions, where most of the participants selected “Agree” or “Strongly agree” answers. Through cross-validation of these questions, we observed a response bias, and, therefore, have excluded these questions from the survey analysis. We believe that the response bias was the result of three problems: 1) Some questions were too difficult to understand and participants selected any answer to continue; 2) the question text was inherently biased; and 3) some participants tended to select only positive answers (acquiescence bias). We will reflect on this issue in the Discussion-section.

Out of a total of 45 started surveys, we registered 35 completed surveys. While two participants were not able to finish the survey due to technical reasons, other participants accidentally left the survey while in the process of filling it out. Since the application only saves the question results of a finished section, these participants had to start again from the last started section. Therefore, even though the application statistics report on the number of started surveys, the application does not register the number of times a participant re-started a survey section and does not save a partially responded section. Hence, it is not possible to statistically identify the number of times participants filled out the questions of repeated sections. Based on observations, around one-third of the participants reported to the survey team that they accidentally left the survey and inquired the team how to get back to continue filling the survey. Re-starting the survey section might have resulted in a learning curve that was not intended by the survey design. For the analysis, only the survey data from completed surveys were used.

## 4. Experiment 2: Colombian case study

Based on our experiences from the first experiment (Ugandan Case Study), we slightly adjusted the study design for the second experiment (Colombian Case Study). For the second experiment, we applied the same analysis as for the first experiment. In the sections that follow, we will first outline the differences in our methodology and will then proceed to present the results of the second experiment.

### 4.1 Participant recruitment

Participants were recruited through Fundación Ecohabitats, a non-governmental organization, which works together with farmers to sustainably improve their agricultural practices. Participant recruiting was done through phone contacts with their collaborating farmers in the Popayan region. Again, we asked to invite at least 35 female and male farmers ranging from ages 18 to 65 years, with Spanish reading and writing literacy and familiarity with smartphones.

In total, 39 participants were recruited, of which two participants did not finish the survey due to technical issues. The mean age was 44 (SD = 12.99). 16 women and 21 men participated (almost achieving a gender balance), of which 35.1% were owners of a smartphone (as for the Ugandan Case Study, all participants had previously used a smartphone). How comfortable the participants felt using a smartphone was rated on a 5-point-Likert scale (where low values equal less comfort), with a resulting mean of 2.86 (SD = 1.44). The same was conducted with map use comfort, leading to a mean of 3 (SD = 1.52). Table 4 shows a detailed view of the participants’ profile data.

**Table 4:** Participants’ characteristics.

Age	Mean: 43.76
	SD: 12.99
	Min: 20
	Max: 65
Gender	Female: 16
	Male: 21
Education	Primary school: 23
	Secondary school: 13
	Undergraduate: 1
Smartphone ownership	Yes: 35.1%
	No: 64.9%
How comfortable participants felt using a smartphone 1 (min)— 5 (max)	Mean: 2.86
	SD: 1.44
How frequently participants used a smartphone 1 (min)—3 (max)	Mean: 2.22
	SD: 0.75
Whether participants used the smartphone for applications other than Social Media	Yes: 56.8%
	No: 43.2%
Whether participants had prior experiences with using a map	Yes: 67.6%
	No: 32.4%
How comfortable participants felt using a map 1 (min)—5 (max)	Mean: 3
	SD: 1.52
How frequently participants used a map 1 (min)—3 (max)	Mean: 1.22
	SD: 0.42

### 4.2 Materials

**Fig 5:**
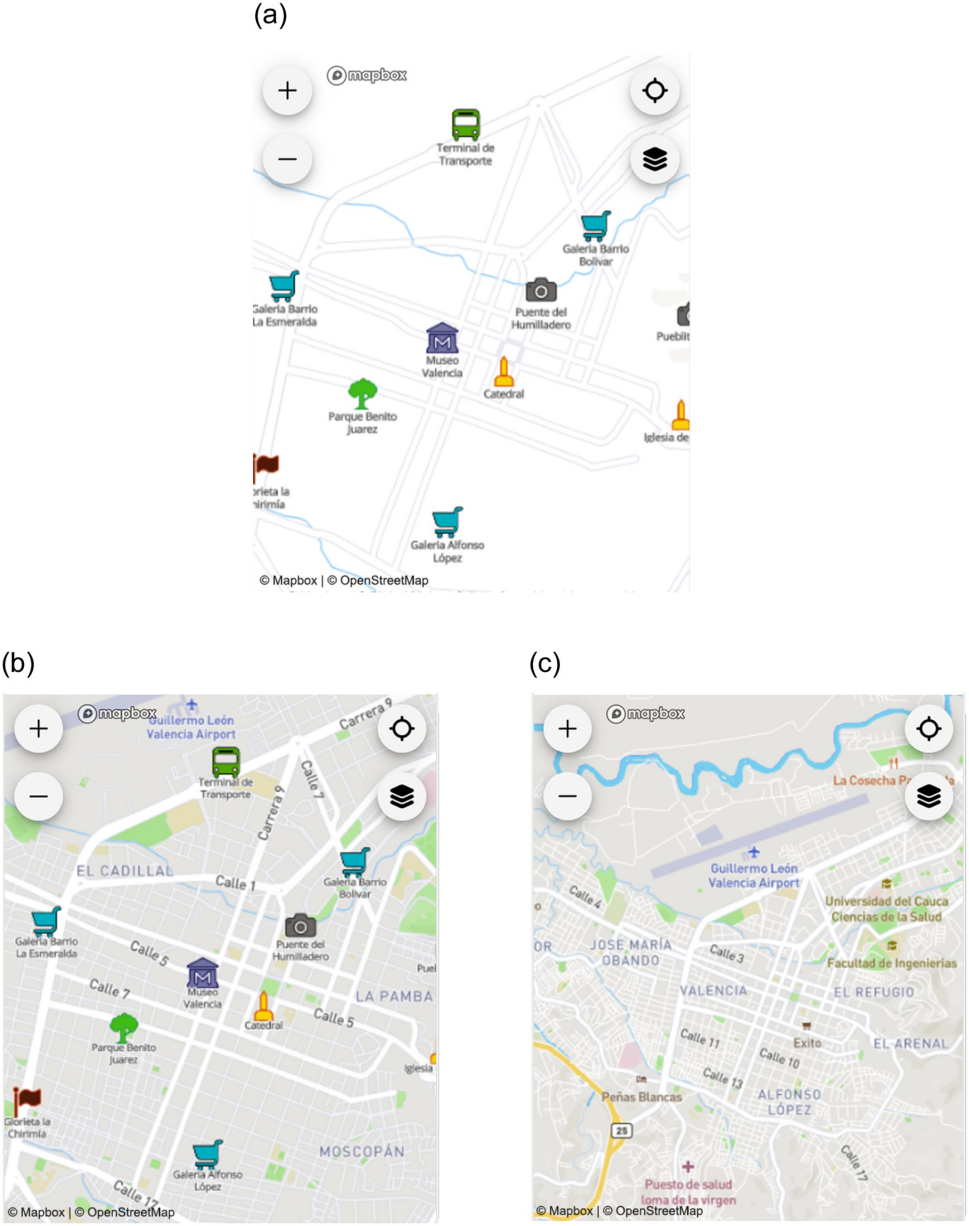
Types of base maps with a) Landmark map, b) Simple map, and c) Mapbox Streets base map (note that the figures use map data from Mapbox and OpenStreetMap and their data sources; also see mapbox.com/about/maps and openstreetmap.org/copyright).

We used the same map design variations as for the Ugandan Case Study but focused the map on the city of Popayan, Colombia (Fig 5a–5c). Each map design variation was coupled with a question to prompt participants’ interaction. S2 Table provides the full set of the 24 questions for each map design variation.

### 4.3 Survey structure and procedure

While for the Ugandan Case Study each participant received a randomized subset of twelve map design variations, we increased this number for the Colombian experiment to 15 variations to have a higher total number of responses. The more responses for each map design variation, the better we can estimate the effects of the different map elements and participant characteristics.

Additionally, we made some adjustments to the survey structure. First, instead of only showing tutorial videos, as had been done for the Ugandan Case Study, we opted to combine these videos with three pre-test map design variations and a respective question. Based upon our prior observations in the Ugandan Case Study, we realized that the participants were confused by the tutorial videos that showed interactions with the map design variations. Instead of just watching the videos, they expressed a desire to interact with the map as part of the pre-test activity. Hence, we combined the tutorial videos with three pre-test map design variations, where we showed the participants all three types of map-reading tasks, base map styles, and interactivity variants (corresponding to the map design variations 7, 11, and 22, see S2 Table).

Second, as done in the Ugandan Case Study, after posing each map design variation, we asked about participants’ self-rated comfort and confidence. Since we observed confusion on these questions and a resulting strong response bias in the Ugandan Case Study, we modified the self-rating questions for the Colombian Case Study (Table 5).

**Table 5:** Follow-up questions after map design variation (*this question was included for evaluating a possible response bias).

#	Question:	Choices:
1	Were you able to successfully solve the map-reading task?*	Yes
		No
2	How did you feel when solving the map-reading task?	Comfortable
		Uncomfortable
		Indifferent
		I did not respond
3	Do you think your solution to the map-reading task was…	Correct
		Incorrect
		I am not sure

Third, due to the detected strong response bias in the base map preference questions of the last survey part of the Ugandan participants, for the Colombian Case Study, we simplified this section and only compared the three base map styles. We presented three questions, each showing two maps to compare: Landmark map and Simple map; Simple map and Mapbox Streets; Landmark map and Mapbox Streets. For each comparison question, we asked the participants whether they preferred a base map style (and which one) or if they liked both.

The procedure of the surveys was the same as in the first experiment. We carried out the experiment in a school hall in the village Vereda Los Cerrillos and the participants were compensated with lunch (equivalent value of 15,000 COP).

### 4.4 Results

In the following, we will describe the results of the descriptive analysis and the regression models of the Colombian experiment.

#### 4.4 1 Task success, comfort, and confidence ratings

On average, each map design variation was used 23.12 times (see also the frequency of each map design variation in S2 Table). The mean task success of the map design variations was 61%. The mean self-rated comfort and confidence amounts to 88% and 84%, respectively.

**Fig 6:**
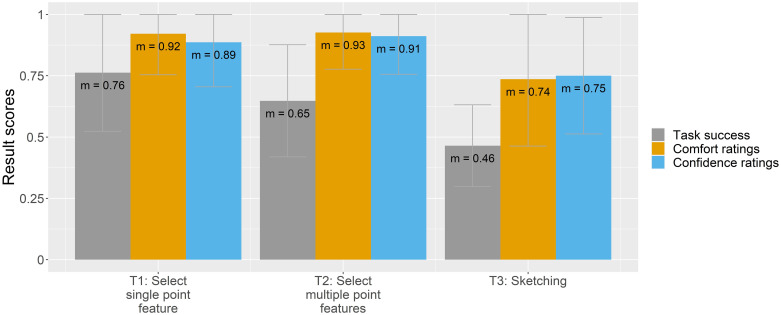
Task success, comfort, and confidence ratings by map-reading tasks.

**Fig 7:**
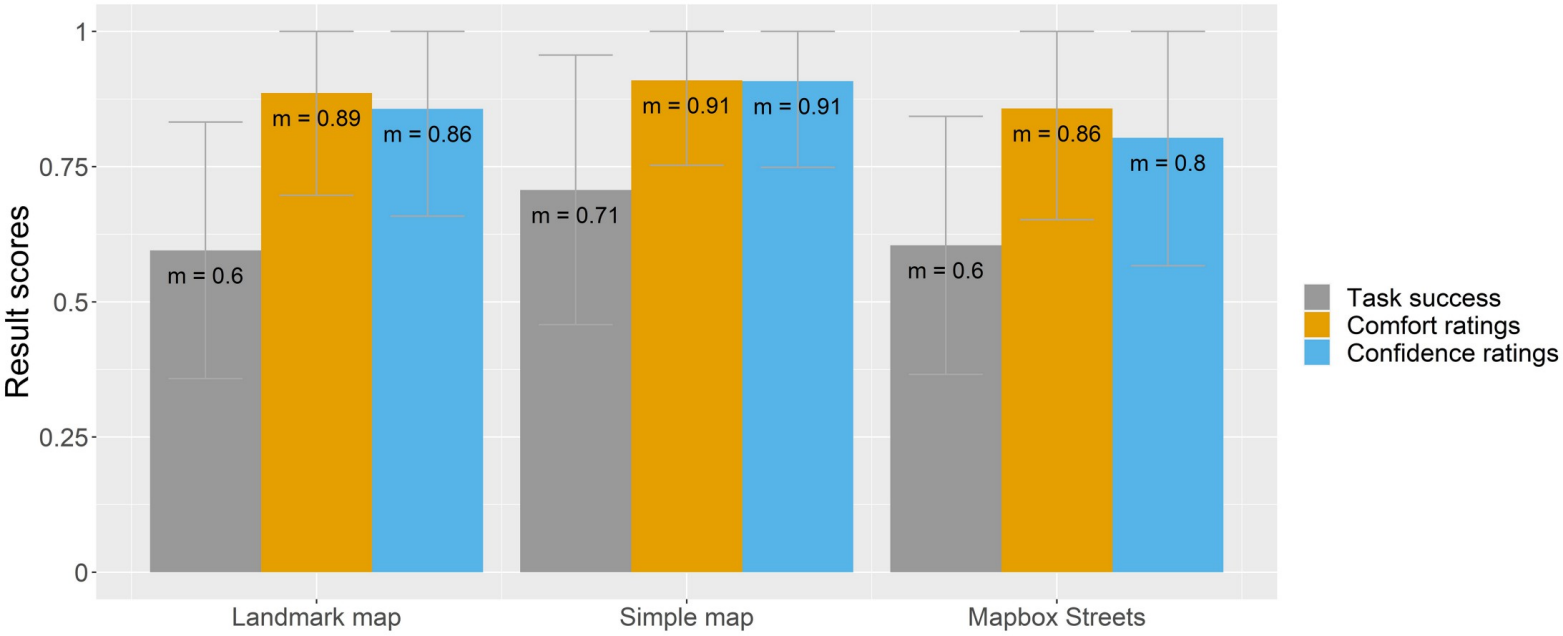
Task success, comfort, and confidence ratings by base map style.

**Fig 8:**
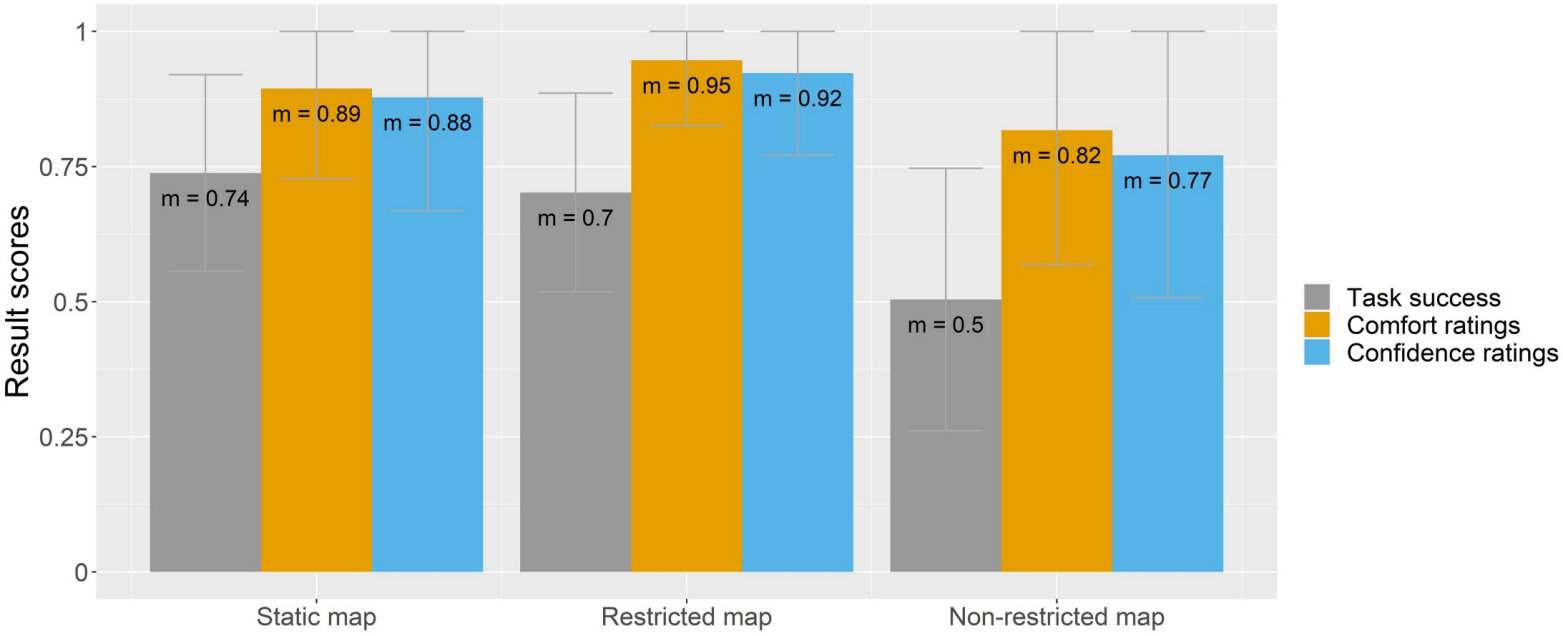
Task success, comfort, and confidence ratings by interactivity variant.

To understand how participants felt completing each map-reading task, base map style, and interactivity category, the following figures (Figs 6–8) compare all three result scores (task success, comfort, and confidence ratings).

Fig 6 shows the result scores by map-reading tasks. The highest task success yields the map-reading tasks to select a single point feature (T1) and to select multiple point features (T2) with 76% and 65%, respectively. For both tasks, the self-rated comfort and confidence were similarly high, ranging around 90%. In contrast, the lowest result scores are assigned to the map-reading task where we asked participants to sketch on the map (T3).

For the base map category (Fig 7), the highest result scores are assigned to the Simple map, with a task success of 71% and comfort and confidence ratings of 91%. With slightly lower scores, the Landmark map and Mapbox Streets base map show similar scores, with a task success of 60% and comfort and confidence ratings ranging between 80 and 89%.

A slightly different trend is visible in terms of the base map preference questions that were posed in the last survey section. When we asked the participants to compare the Landmark map to the Simple map, 42% of the participants preferred the Landmark map, 29% of the participants chose the Simple map, and 29% liked both base map styles. For the comparison between the Simple map and Mapbox Streets base map, 34% of the participants chose the Simple map and 42% Mapbox Streets (24% liked both equally). And lastly, 53% of the participants preferred the Landmark map over the Mapbox Streets base map (18%), and 29% liked both base map styles.

Finally, Fig 8 shows the result scores by each interactivity variant. While the highest task success scores (74%) are assigned to the static map version, participants’ comfort and confidence ratings were slightly higher for the restricted map variant (between 92 and 95%). The non-restricted map yields the lowest result scores.

#### 4.4.2 Task success, comfort, and confidence ratings by map design variation

**Fig 9:**
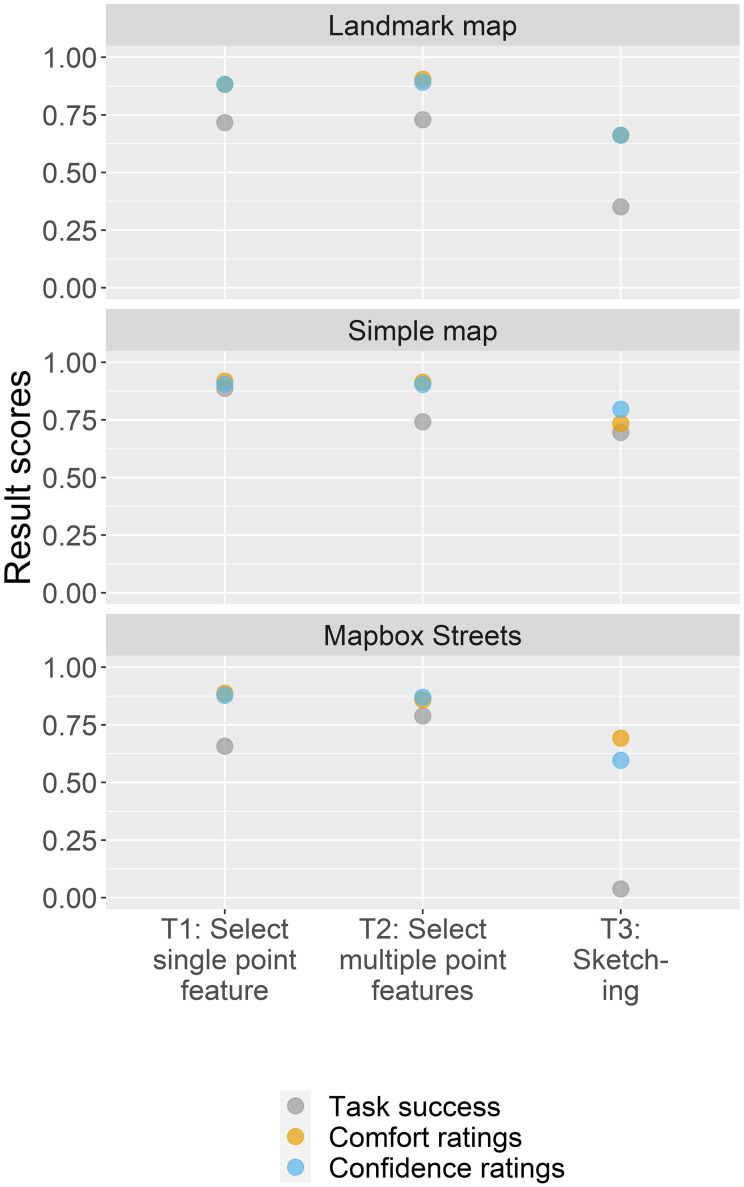
Task success, comfort, and confidence ratings of map design combinations.

**Fig 10:**
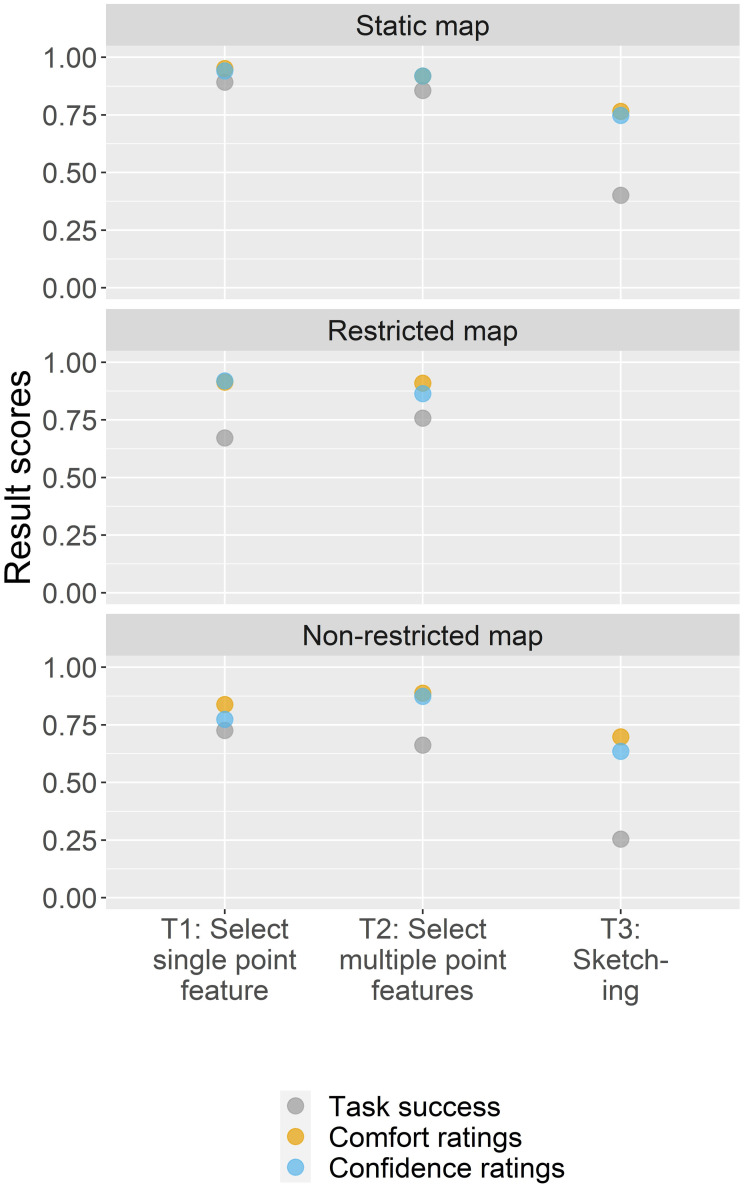
Task success, comfort, and confidence ratings of map design combinations.

**Fig 11:**
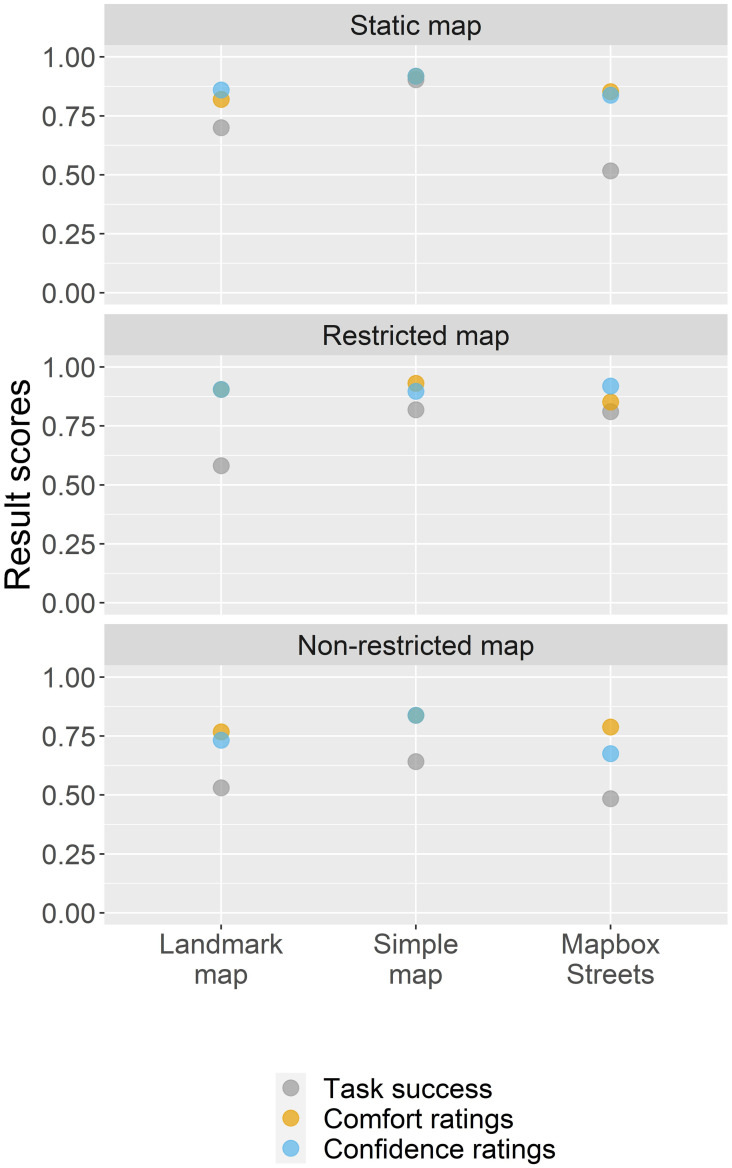
Task success, comfort, and confidence ratings of map design combinations.

For evaluating the result scores for the variations of map design elements, we further calculated result scores for the map-reading tasks in regard to the base map styles (Fig 9) and interactivity variants (Fig 10), as well as the base map styles in regard to the interactivity variants (Fig 11). The result scores show that the map-reading tasks to select a single point feature (T1) and to sketch on the map (T3) were aided when coupled with a Simple map. For the second map-reading task to select multiple point features (T2), the scores are rather similar across base map styles. In general, all map-reading tasks yield higher result scores when coupled with a static map (this is also statistically supported for T1 and T2 where the task success was negatively correlated larger interactivity complexities with p < 0.05 * and p < 0.01 ***, respectively; participants comfort and confidence for T1 were negatively correlated with larger interactivity complexities with p < 0.05 * and p < 0.01 **, respectively). However, the restricted and non-restricted maps were rather similar in their scores. We further find that the Landmark map worked best when combined with a static interactivity version, the Simple map worked best either with the static or restricted variant, and Mapbox Streets was aided when combined with the restricted variant.

#### 4.4.3 Logistic mixed-effects regression analysis

For analyzing the relation between user characteristics, map design elements, task success, and participants’ self-rated comfort and confidence, we built three logistic mixed-effects regression models.

Table 6 shows the model outcome for each dependent variable (task success, comfort, and confidence ratings) by displaying the SBC and the p-values of the variables. For the first model with task success as the dependent variable, the map-reading tasks are depicted as significant (p < 0.01 **), indicating that the task success decreases with the increase in map-reading task category (from first to third). This trend is followed with the interactivity variants (p < 0.05 *), meaning that an increase in interactivity complexity leads to a decrease in task success. Both the comfort and confidence ratings are highly positively correlated with task success (p < 0.001 ***).

**Table 6:** Regression models with SBC and p-value with task success, comfort, and confidence ratings as the dependent variables (see S4–S6 Tables for odds ratios of each model).

#	Variables	Task success	Comfort ratings	Confidence ratings
1	Task success		2.44 ***	1.98 ***
2	Comfort ratings	2.16 ***		3.65 ***
3	Confidence ratings	1.74 ***	3.01 ***	
4	Map-reading tasks	-2.22 **	-0.07	-0.27
5	Base map styles	-0.23	0.11	-0.29
6	Interactivity variants	-1.43 *	0.31	-0.74
7	Time spent on task	0.04	-0.99.	-0.33
8	Age	0.07	-1.59	-0.27
9	Gender (male/female)	-0.29	-1.08	2.00 ***
10	Education	-1.02.	-0.62	0.14
11	Owner of smartphone (yes/no)	0.40	-0.36	2.76
12	Smartphone use comfort	-0.06	0.40	-0.59 *
13	Smartphone use frequency	1.64.	-1.04	0.14
14	Smartphone application use other than social media (yes/no)	-0.71.	-0.02	-0.11 *
15	Map use experience (yes/no)	-0.46	2.56 *	-0.25
16	Map use comfort	0.40.	0.12	-0.33
17	Map use frequency	-0.73	-3.04	-1.79

For the second model with the comfort ratings as the dependent variable, the task success and participants’ confidence are positively correlated with the comfort ratings (p < 0.001 ***). In addition, participants having used a map before were more likely to feel comfortable when interacting with the map design variations (p < 0.05 *).

Lastly, for the third model with confidence ratings as the dependent variable, the task success and comfort ratings are positively correlated with the confidence ratings of the participants (p < 0.001 ***). Further, men were more likely to indicate that they felt confident in using the map design variations (p < 0.001 ***). Interestingly, participants having indicated to feel comfortable in using smartphones and also using smartphones for various purposes (other than social media) were less likely to feel confident in using the map design variations (p < 0.05 *).

#### 4.4.4 Observations

While participants filled out the survey, many distractions were presented. Firstly, some participants did not honor their scheduled time slots, which resulted in many participants arriving at the same time and some of them had to wait. Secondly, some participants came to the study center with family members, which further resulted in a crowded atmosphere and, therefore, generated some distractions for the participants.

Based on our direct observation, many participants had little experience in smartphone use and consulted other participants or their accompanying family members. Even though we asked participants to complete work on their own, we were not able to prevent the participants from working with each other in some instances. This might have affected participants’ performance in the survey.

Out of 45 started surveys, we registered 37 completed surveys. While two participants were not able to finish the survey due to technical issues, we observed other participants accidentally closing the survey or leaving the application (as we observed with some Ugandan participants). As for the Ugandan Case Study, we are not able to provide statistics about question and section repetitions, but the survey team reported having to help around 25% of the participants to re-start a survey section.

We assume that these described factors are sources of potential bias in our survey results. Although we changed the self-rated questions based on the lessons learned from the Ugandan Case Study, through cross-validation we still observed a potential acquiescence bias.

## 5. Discussion

Even though more Ugandan participants indicated owning a smartphone and also indicated a higher average value of self-rated smartphone and map use experience in comparison to Colombian participants, the overall task success was lower. This might be explainable by Colombian participants generally being more exposed to using smartphones (even though they were not owning one themselves) as well as the detected response bias and resulted in a different set of influencing variables on the task success. In the Ugandan Case Study, the average task success amounts to 51%. Comparing the task success of the different map-reading tasks, base map styles, and interactivity variants, we detected the most significant variation between the interactivity variants, where higher freedom in interactivity showed a lower task success. This was further supported by the regression analysis, with a higher degree of interactivity being negatively correlated with the task success, and is also in accordance with findings by Vincent et al. [20].

In contrast, Colombian participants had a higher task success with 61%, and the most variation in task success between the map design variations was detected by the map-reading tasks, which was also confirmed by the regression model. This trend was further supported by the confidence and comfort ratings, which were lowest for the third map-reading task, where participants were asked to sketch on the map (T3). In general, the comfort and confidence ratings were always higher than the task success, which is in concurrence with other study findings and regarded as participants’ overconfidence [44, 45]. However, we observe that the comfort and confidence ratings show similar trends, compared to task success, in result scores across map design variations.

While for the Ugandan participants the interactivity was the most correlated variable with the task success, we noted that the task success also decreased by the complexity of the map-reading tasks. For the Colombian participants, this was the other way around, where the map-reading task was the dominant influencer on the task success, but a variation in task success was also detected by higher freedom in interactivity. The comfort and confidence ratings also decreased for the non-restricted interactivity variant, where participants were free to zoom and pan around on the map.

Hence, we assume that for participants that are not much used to working with smartphones and map interfaces, the degree of interactivity and the map-reading tasks may play a significant role. This is also reflected by the comfort and confidence ratings (for Colombian participants), where ratings were assigned to the map-reading task to sketch on the map (T3) and the non-restrictive interactivity variant.

There is not a clear pattern regarding the task success that pertains to the use of different base map styles. This is also supported by the comfort and confidence ratings that were similar for all base map styles. However, for both Ugandan and Colombian participants, the Simple map showed a higher task success. A general tendency towards highlighted landmarks is visible since the Mapbox Streets base map was less well-received than the Simple map (which was primarily modified by highlighting the landmarks). This is presumably due to the decrease in cognitive load when landmarks were highlighted on the map [35].

In terms of map design variations, the task success of all variations was higher when combined with a static map. Nevertheless, the map-reading task to select multiple point features (T2) and the Simple map also showed higher task success scores for the restricted (and partially non-restricted) map variant. In general, T2 yielded high result scores across all base map styles. The rather difficult map-reading task to sketch on the map (T3) was aided when coupled with a static Simple map. We further observed improved result scores for the static Landmark map, static or restricted Simple map, and restricted Mapbox Streets base map.

Participants’ age, gender, educational level, and the time spent on each map design variation were not important factors for influencing the task success. Some smartphone and map use variables were detected in some regression models as influencing factors. However, no measured participant characteristic seems to be a particularly relevant impactor on the task success.

In spite of a wide range of practical challenges that we faced throughout the study, we were still able to depict the differences between study areas and their participants. While participants were all primarily smartphone use inexperienced farmers, there was still a variation in how the participants responded to the survey. This is an important insight to validate the usefulness of adapting the map design to user characteristics and contexts of use. Hence, with the results from this research, we are able to understand which map element tended to better support the farmers who are the target of collaborative agricultural applications. Reducing map-reading task and interactivity complexities will substantially improve the UX for these farmers. Since our farmers consisted primarily of less experienced smartphone and map users, our findings may also be helpful for those designing map applications that target less-experienced smartphone and map users in general.

Our work helps establish some basic principles for designing map adaptations [22]. This is especially relevant in the agricultural domain since farmers are considerably benefiting from informed decision-making processes but are challenged using mobile applications to receive this information [13, 46]. To achieve matching the application design and map design to the users’ requirements, the map design adaptation should help defining which user contextual information is most relevant to consider. Adaptive or adaptable mobile map applications will be of increasing interest in the future to address the diversity of user groups and agricultural mobile map application use purposes. Research on application use and user diversity is of increasing interest and its relevance for usability and UX research is undeniable. Regression analyses, as carried out in our study, will help in understanding the factors that should influence the (adaptation) of the map design of mobile map applications. The results of this study underline the usefulness of this approach to relate user contextual information to the map design and to prioritize certain context and map design elements.

The kind of empirical studies we carried out in this research is necessary to analyze how user context modifies the UX of map design elements. However, as we did not conduct the survey in a laboratory environment, but with a variety of user types and situations, we were presented with challenging conditions. While we were unable to control for every contextual factor as it would have been possible in a laboratory environment, we were able to understand much better the users, their smartphone use situations, and their smartphone use behavior in these situations [47]. Thereby, we saw it important to conduct two independent case studies because we were able to adjust the survey design after the first experiment. We especially realized that approaches like the SUS [40], even though widely used, are not helpful in the study areas where we conducted our experiments. In the Ugandan Case Study, participants simply did not understand the phrasing of the SUS statements. Therefore, we argue that usability/UX heuristics have to be reviewed for their effectiveness with a diversity of study participants, especially in those or similar study areas as we worked with in our research.

In terms of the encountered challenges of the study, participants showed a variety of difficulties in understanding the questions, which the map design variations featured, due to reading comprehension issues or the phrasing of the questions. As previously mentioned, another challenge was the acquiescence bias of the self-rating and preference questions. Some of the data we were particularly interested in for evaluating not only the task success but also the UX (through the comfort and confidence ratings) had to be excluded from the analysis. The same problem was detected with the questions about self-rated smartphone and map use experiences. In the literature, three ways to avoid acquiescence bias are commonly discussed: using a balanced Likert scale [48, 49], using item-specific question choices [49], and statistically correcting the bias post-hoc [48, 49]. As significant differences exist between population groups, cultures, and countries [48, 50], we identified with our participant groups that we had to use very simple phrases and words suited to the participants’ language. Triangulation of multiple questions that ask the same item to investigate but in different ways might help as well. In addition, instead of asking about participants’ self-evaluation, it might be useful to pose more objective and measurable questions (such as smartphone use frequency instead of a self-evaluated smartphone use experience). We assume that questions about the participants’ self-evaluation and preferences, in general, may not provide a reliant information source with the type of participants we included in the study. The detected inclination towards responding very positively may not be overcome by posing the questions as neutrally as possible, which is why it is important to evaluate suggested or alternative ways to study the preference, and self-evaluation factors.

## 6. Conclusion

In this study, we evaluated map design elements for collaborative mobile mapping applications for agricultural users. Through the survey module of GeoFarmer, we tested 24 map design variations, which combined three map-reading tasks, three base map styles, and three interactivity variants. We evaluated these elements with 72 coffee farmers in two different case studies on two different continents in the countries of Uganda and Colombia.

The principal factor for impacting the task success of our farmer participants (who consisted mostly of lower experience levels in smartphone and map use) was the freedom of interactivity and the complexity of the map-reading tasks. The base map styles played a minor role in the UX of the participants.

While we intended to also analyze the UX of the participants by evaluating their confidence and comfort ratings, we encountered a response bias, which posed a limiting factor for the data analysis. Further, most of the participants had a similar set of user characteristics, which is why these factors did not have the power to explain differences in performance and ratings.

The findings of our study demonstrate that including maps in collaborative mobile interfaces for farmers is possible as long as their design matches users’ characteristics and contexts of use. In the future, farmers will have increasing opportunities to use smartphone and collaborative applications. Because agricultural information is usually grounded by a geospatial component, implementing adaptive elements in collaborative mobile mapping applications will play an important role in supporting decision-making by farmers.

Our results will help other investigators to understand the pitfalls and opportunities presented by empirical studies of map design elements with users coming from diverse circumstances. Although our work faced many practical hurdles, these were all due to the fact that we chose to work with participants in complex environments and with varying degrees of technological experience. Had we chosen to study university students in a developed country, we no doubt could have more completely controlled all external factors, but we would have learned very little then about what it will actually take to adapt mapping interfaces to the places and people who really need them. Our findings may not only serve to give recommendations for adaptive mobile map elements to support farmers but may also potentially be extended to other user audiences that have less experience with smartphones and/or map use. Therefore, our work helps establish some key observation points for user context-based map design adaptations.

Future research is suggested to build on what we have begun here. For example, future studies could focus on testing adaptable map design elements that were not included in this research. Among these, testing a more fine-grained set of map-reading tasks would help to pinpoint in a more detailed way which map-reading tasks can be posed for which user type. In this study, we did not separate for the specific analytical map-reading task, such as selecting points based on distances or based on specific attributes. This, however, would be useful to understand the differences in UX between these activities. Additional map-reading tasks, such as data layer comparisons, creating points, and inputting respective point information or images, instead of just selecting a point, and interacting with line or polygon feature types, might be useful to evaluate. Also, satellite imageries are especially relevant for farmers since base map styles, such as Mapbox Streets, are mostly not detailed enough for the areas where farmers live or have their agricultural land. Testing this kind of base map in combination with other adaptive map design elements, such as the map-reading tasks, interactivity complexity, or time pressure (which is usually encountered when outside on the field), would help add more data points to our understanding of how user context modifies map design UX. We also recommend that future studies collect usability evaluation data from a larger number of participants, resulting in a larger sample size than of the present study.

Future research could also evaluate how to better assess user experiences in contexts where literacy and technology access may be challenging, including assessing users’ comfort or confidence. In our research, we noted multiple instances in which there was observable bias pertaining to these factors.

The development of user context-based map adaptations for collaborative agricultural mobile applications is an important piece in advancing research about adaptive map interfaces and the diversity of application users and purposes of use. This will be increasingly important in the future since the technological development of interface design, in general, is now able to implement advances made in the area of adaptive interfaces. This development will aid the design of effective collaborative mobile agriculture applications used by diverse farmer user groups with diverse use purposes to support them by accessing relevant information and supporting them in making smart and informed decisions.

## Supporting information

S1 Table Map design variation with a respective question and answer frequency—Ugandan case study. (DOCX)

S2 Table Map design variation with a respective question and answer frequency—Colombian case study. (DOCX)

S3 Table Odds ratio and confidence intervals for regression model of Table 3. (DOCX)

S4 Table Odds ratio and confidence intervals for regression model of Table 6 with task success as dependent variable. (DOCX)

S5 Table Odds ratio and confidence intervals for regression model of Table 6 with comfort ratings as dependent variable. (DOCX)

S6 Table Odds ratio and confidence intervals for regression model of Table 6 with confidence ratings as dependent variable. (DOCX)
