# Supplementary material for: The impact of user characteristics of smallholder farmers on user experiences with collaborative map applications
Source: PLoS One. 2022 Mar 2;17(3):e0264426. doi: 10.1371/journal.pone.0264426 (PMC8890669; doi:10.1371/journal.pone.0264426)
Supplement: S1 Table — (DOCX) [file pone.0264426.s001.docx]

**S 1 Table:** **Map design variation with a respective question and answer frequency – Ugandan Case Study**

| # | Map-reading task: | Question: | Variation: | Fre-quency: |
| --- | --- | --- | --- | --- |
| 1 | Selecting single point feature | Can you identify the Central market? Please select. | Static Landmark map | 16 |
| 2 |  | Can you identify the yellow marker? Which is the closest market to that position? | Restricted Landmark map | 17 |
| 3 |  | Can you identify the Mutukula market? Please select the closest hospital to that market? | Non-restricted Landmark map | 18 |
| 4 |  | Can you identify the Bugwere market? Please select. | Static Simple map | 17 |
| 5 |  | Can you identify the yellow marker? What is the closest market to it? | Restricted Simple map | 18 |
| 6 |  | Can you identify the Namatala market? Please select. | Non-restricted Simple map | 19 |
| 7 |  | Can you identify the forest close to the market you see on the map? Please select. | Static Mapbox Streets | 18 |
| 8 |  | Which market is furthest away from the yellow marker? Please select. | Restricted Mapbox Streets | 18 |
| 9 |  | Which is the closest hospital to the Kumi Road Market? Please select. | Non-restricted Mapbox Streets | 18 |
| 10 | Selecting multiple point features | The green areas are agricultural land. Which two fields are the biggest? Please select two. | Static Landmark map | 18 |
| 11 |  | Can you identify all the markets on the map? Please select. | Restricted Landmark map | 17 |
| 12 |  | Can you identify all the schools on the map? Please select. | Non-restricted Landmark map | 17 |
| 13 |  | The areas represent land use. Can you select all markers on the green areas? | Static Simple map | 18 |
| 14 |  | Which of these markers are in parks or green areas? Please select at least two. | Restricted Simple map | 19 |
| 15 |  | Can you identify the yellow marker? Please select the two closest markets to it. | Non-restricted Simple map | 18 |
| 16 |  | Can you identify the two markers that are located in the largest parks/green areas? Please select. | Static Mapbox Streets | 16 |
| 17 |  | Can you identify the Mutukula market and Central Market? Please select both. | Restricted Mapbox Streets | 18 |
| 18 |  | The Mbale Regional Referral Hospital is in the center of the city. Please select the two closest schools to it. | Non-restricted Mapbox Streets | 16 |
| 19 | Sketching | In your opinion, which area on the map belongs to Mt. Elgon? Please draw on the map! | Static Landmark map | 17 |
| 20 |  | Where is Mt. Elgon located? Please move the map and draw the area. | Non-restricted Landmark map | 17 |
| 21 |  | In your opinion, where are coffee-growing areas? Please draw on the map! | Static Simple map | 18 |
| 22 |  | In your opinion, where are coffee-growing areas? Please move the map and draw the area. | Non-restricted Simple map | 17 |
| 23 |  | Where do you think are the best areas to grow coffee? Please draw on the map! | Static Mapbox Streets | 18 |
| 24 |  | Where do you think are the best areas to grow coffee? Please move the map and draw the area. | Non-restricted Mapbox Streets | 17 |
