# Supplementary material for: The impact of user characteristics of smallholder farmers on user experiences with collaborative map applications
Source: PLoS One. 2022 Mar 2;17(3):e0264426. doi: 10.1371/journal.pone.0264426 (PMC8890669; doi:10.1371/journal.pone.0264426)
Supplement: S2 Table — (DOCX) [file pone.0264426.s002.docx]

**S 2 Table: Map design variation with a respective question and answer frequency – Colombian Case Study**

| # | Map-reading task: | Question: | Variation: | Fre-quency: |
| --- | --- | --- | --- | --- |
| 1 | Selecting single feature | Can you identify the cathedral in the city center? Please select. | Static Landmark map | 24 |
| 2 |  | Which of the following markets is closest to the bus terminal: Galeria Barrio La Esmeralda or Galeria Barrio Bolivar? | Restricted Landmark map | 26 |
| 3 |  | Can you identify Glorieta la Chirimía? Please select. | Non-restricted Landmark map | 22 |
| 4 |  | Can you identify the bus terminal? Please select. | Static Simple map | 23 |
| 5 |  | Which of the following markets is closest to the cathedral: Galeria Barrio La Esmeralda or Galeria Barrio Bolivar? | Restricted Simple map | 19 |
| 6 |  | Can you identify the Galeria Barrio Bolivar? Please select. | Non-restricted Simple map | 22 |
| 7 |  | Can you identify the park Caldas? Please select. | Static Mapbox Streets | 28 |
| 8 |  | Which of the following markets is furthest away from the bus terminal: Galeria Alfonso López or Galeria Barrio Bolivar? | Restricted Mapbox Streets | 24 |
| 9 |  | Can you identify the park El Morro? Please select. | Non-restricted Mapbox Streets | 18 |
| 10 | Selecting multiple features | Which of the parks are the two biggest ones? | Static Landmark map | 24 |
| 11 |  | Which two markets are closest to the Glorieta La Chirimía? | Restricted Landmark map | 23 |
| 12 |  | Can you identify all the markets and malls? Please select. | Non-restricted Landmark map | 23 |
| 13 |  | Can you identify the Puente del Humilladero and the Park Benito Juarez? Please select. | Static Simple map | 23 |
| 14 |  | Which two parks are the closest to the Market Barrio Bolívar? | Restricted Simple map | 25 |
| 15 |  | Can you identify all the churches on the map? Please select. | Non-restricted Simple map | 23 |
| 16 |  | Can you identify the Hospital Susana Lopez de Valencia and the Glorieta la Chirimía? Please select. | Static Mapbox Streets | 23 |
| 17 |  | Can you identify the parks El Morro and Alfonso Lopez? Please select. | Restricted Mapbox Streets | 23 |
| 18 |  | Next to the Punte del Humilladero is a park and a museum. Please select both. | Non-restricted Mapbox Streets | 23 |
| 19 | Sketching | Where do you think is the center of Popayan located? Please draw on the map. | Static Landmark map | 23 |
| 20 |  | Where do you think is the center of Popayan located? Please move the map and draw the area. | Non-restricted Landmark map | 21 |
| 21 |  | Where do you think is the area of park El Morro? Please draw on the map. | Static Simple map | 21 |
| 22 |  | Where do you think is the area of park El Morro? Please move the map and draw the area. | Non-restricted Simple map | 22 |
| 23 |  | Where do you think is the center of Popayan located? Please draw on the map. | Static Mapbox Streets | 26 |
| 24 |  | Where do you think is the center of Popayan located? Please move the map and draw the area. | Non-restricted Mapbox Streets | 26 |
