# Supplementary material for: The impact of user characteristics of smallholder farmers on user experiences with collaborative map applications
Source: PLoS One. 2022 Mar 2;17(3):e0264426. doi: 10.1371/journal.pone.0264426 (PMC8890669; doi:10.1371/journal.pone.0264426)
Supplement: S3 Table — (DOCX) [file pone.0264426.s003.docx]

**S 3 Table: Odds ratio and confidence intervals for regression model of Table 3**

| **#** | **Variables** | **Odds ratio** | **Confidence interval** | |
| --- | --- | --- | --- | --- |
|  |  |  | **2.5%** | **97.5%** |
| 1 | Map-reading tasks | 0.69 | 0.43 | 1.13 |
| 2 | Base map styles | 0.73 | 0.46 | 1.17 |
| 3 | Interactivity variants | 0.46 | 0.30 | 0.72 |
| 4 | Time spent on task | 0.84 | 0.63 | 1.11 |
| 5 | Age | 0.77 | 0.51 | 1.15 |
| 6 | Gender (male/female) | 1.88 | 0.64 | 5.49 |
| 7 | Education | 0.87 | 0.56 | 1.36 |
| 8 | Owner of smartphone (yes/no) | 1.04 | 0.33 | 3.25 |
| 9 | Smartphone use comfort | 0.96 | 0.54 | 1.69 |
| 10 | Smartphone use frequency | 0.69 | 0.31 | 1.51 |
| 11 | Smartphone application use  other than social media (yes/no) | 2.63 | 0.95 | 7.22 |
| 12 | Map use experience (yes/no) | 1.06 | 0.46 | 2.44 |
| 13 | Map use comfort | 1.11 | 0.75 | 1.64 |
