# Supplementary material for: The impact of user characteristics of smallholder farmers on user experiences with collaborative map applications
Source: PLoS One. 2022 Mar 2;17(3):e0264426. doi: 10.1371/journal.pone.0264426 (PMC8890669; doi:10.1371/journal.pone.0264426)
Supplement: S4 Table — (DOCX) [file pone.0264426.s004.docx]

**S 4 Table: Odds ratio and confidence intervals for regression model of Table 6 with task success as dependent variable**

| **#** | **Variables** | **Odds ratio** | **Confidence interval** | |
| --- | --- | --- | --- | --- |
|  |  |  | **2.5%** | **97.5%** |
| 1 | Comfort ratings | 4.42 | 2.22 | 8.81 |
| 2 | Confidence ratings | 5.03 | 1.96 | 12.91 |
| 3 | Map-reading tasks | 0.25 | 0.10 | 0.58 |
| 4 | Base map styles | 0.87 | 0.40 | 1.89 |
| 5 | Interactivity variants | 0.44 | 0.21 | 0.94 |
| 6 | Time spent on task | 1.02 | 0.76 | 1.36 |
| 7 | Age | 1.04 | 0.56 | 1.90 |
| 8 | Gender (male/female) | 0.75 | 0.37 | 1.53 |
| 9 | Education | 0.40 | 0.15 | 1.06 |
| 10 | Owner of smartphone (yes/no) | 1.30 | 0.47 | 3.61 |
| 11 | Smartphone use comfort | 0.95 | 0.69 | 1.30 |
| 12 | Smartphone use frequency | 1.94 | 0.94 | 4.02 |
| 13 | Smartphone application use  other than social media (yes/no) | 0.48 | 0.23 | 1.03 |
| 14 | Map use experience (yes/no) | 0.58 | 0.18 | 1.86 |
| 15 | Map use comfort | 1.53 | 0.98 | 2.39 |
| 16 | Map use frequency | 0.71 | 0.21 | 2.45 |
