# Supplementary material for: The impact of user characteristics of smallholder farmers on user experiences with collaborative map applications
Source: PLoS One. 2022 Mar 2;17(3):e0264426. doi: 10.1371/journal.pone.0264426 (PMC8890669; doi:10.1371/journal.pone.0264426)
Supplement: S5 Table — (DOCX) [file pone.0264426.s005.docx]

**S 5 Table: Odds ratio and confidence intervals for regression model of Table 6 with comfort ratings as dependent variable**

| **#** | **Variables** | **Odds ratio** | **Confidence interval** | |
| --- | --- | --- | --- | --- |
|  |  |  | **2.5%** | **97.5%** |
| 1 | Task success | 11.53 | 2.74 | 48.41 |
| 2 | Confidence ratings | 55.71 | 15.76 | 196.98 |
| 3 | Map-reading tasks | 0.96 | 0.45 | 2.06 |
| 4 | Base map styles | 1.07 | 0.52 | 2.19 |
| 5 | Interactivity variants | 1.19 | 0.59 | 2.42 |
| 6 | Time spent on task | 0.62 | 0.37 | 1.02 |
| 7 | Age | 0.46 | 0.10 | 2.14 |
| 8 | Gender (male/female) | 0.34 | 0.06 | 1.87 |
| 9 | Education | 0.57 | 0.05 | 6.22 |
| 10 | Owner of smartphone (yes/no) | 0.78 | 0.07 | 8.33 |
| 11 | Smartphone use comfort | 1.49 | 0.69 | 3.21 |
| 12 | Smartphone use frequency | 0.66 | 0.13 | 3.36 |
| 13 | Smartphone application use  other than social media (yes/no) | 0.98 | 0.16 | 5.96 |
| 14 | Map use experience (yes/no) | 21.34 | 1.18 | 386.81 |
| 15 | Map use comfort | 1.13 | 0.37 | 3.46 |
| 16 | Map use frequency | 0.24 | 0.01 | 4.43 |
