# Supplementary material for: The impact of user characteristics of smallholder farmers on user experiences with collaborative map applications
Source: PLoS One. 2022 Mar 2;17(3):e0264426. doi: 10.1371/journal.pone.0264426 (PMC8890669; doi:10.1371/journal.pone.0264426)
Supplement: S6 Table — (DOCX) [file pone.0264426.s006.docx]

**S 6 Table: Odds ratio and confidence intervals for regression model of Table 6 with confidence ratings as dependent variable**

| **#** | **Variables** | **Odds ratio** | **Confidence interval** | |
| --- | --- | --- | --- | --- |
|  |  |  | **2.5%** | **97.5%** |
| 1 | Task success | 7.23 | 2.49 | 21.04 |
| 2 | Comfort ratings | 158.19 | 44.45 | 562.94 |
| 3 | Map-reading tasks | 0.84 | 0.48 | 1.49 |
| 4 | Base map styles | 0.84 | 0.50 | 1.40 |
| 5 | Interactivity variants | 0.66 | 0.39 | 1.11 |
| 6 | Time spent on task | 0.85 | 0.58 | 1.24 |
| 7 | Age | 0.87 | 0.36 | 2.13 |
| 8 | Gender (male/female) | 7.26 | 2.46 | 21.44 |
| 9 | Education | 1.13 | 0.30 | 4.26 |
| 10 | Owner of smartphone (yes/no) | 6.27 | 1.44 | 27.35 |
| 11 | Smartphone use comfort | 0.56 | 0.35 | 0.89 |
| 12 | Smartphone use frequency | 1.06 | 0.42 | 2.63 |
| 13 | Smartphone application use  other than social media (yes/no) | 0.89 | 0.31 | 2.58 |
| 14 | Map use experience (yes/no) | 0.75 | 0.22 | 2.48 |
| 15 | Map use comfort | 0.70 | 0.36 | 1.38 |
| 16 | Map use frequency | 0.43 | 0.12 | 1.54 |
